# Glioblastoma and the search for non-hypothesis driven combination therapeutics in academia

**DOI:** 10.3389/fonc.2022.1075559

**Published:** 2023-01-17

**Authors:** Timothy Johanssen, Laura McVeigh, Sara Erridge, Geoffrey Higgins, Joelle Straehla, Margaret Frame, Tero Aittokallio, Neil O. Carragher, Daniel Ebner

**Affiliations:** ^1^ Target Discovery Institute, Nuffield Department of Medicine, University of Oxford, Oxford, United Kingdom; ^2^ Cancer Research UK Scotland Centre, Institute of Genetics and Cancer, University of Edinburgh, Edinburgh, United Kingdom; ^3^ Edinburgh Cancer Centre, Western General Hospital, Edinburgh, United Kingdom; ^4^ Department of Oncology, University of Oxford, Oxford, United Kingdom; ^5^ Koch Institute for Integrative Cancer Research, Massachusetts Institute of Technology, Cambridge, Department of Pediatric Oncology, Dana-Farber Cancer Institute, Division of Pediatric Hematology/Oncology, Boston Children’s Hospital, Boston, MA, United States; ^6^ Institute for Molecular Medicine Finland (FIMM), HiLIFE, University of Helsinki, Helsinki, Finland; ^7^ Institute for Cancer Research, Department of Cancer Genetics, Oslo University Hospital, Oslo, Norway; ^8^ Centre for Biostatistics and Epidemiology (OCBE), Faculty of Medicine, University of Oslo, Oslo, Norway

**Keywords:** glioblastoma, glioblastoma stem cell, drug target combination, temozolamide, radiotherapy, hypoxia, high throughput screening (HTS)

## Abstract

Glioblastoma (GBM) remains a cancer of high unmet clinical need. Current standard of care for GBM, consisting of maximal surgical resection, followed by ionisation radiation (IR) plus concomitant and adjuvant temozolomide (TMZ), provides less than 15-month survival benefit. Efforts by conventional drug discovery to improve overall survival have failed to overcome challenges presented by inherent tumor heterogeneity, therapeutic resistance attributed to GBM stem cells, and tumor niches supporting self-renewal. In this review we describe the steps academic researchers are taking to address these limitations in high throughput screening programs to identify novel GBM combinatorial targets. We detail how they are implementing more physiologically relevant phenotypic assays which better recapitulate key areas of disease biology coupled with more focussed libraries of small compounds, such as drug repurposing, target discovery, pharmacologically active and novel, more comprehensive anti-cancer target-annotated compound libraries. Herein, we discuss the rationale for current GBM combination trials and the need for more systematic and transparent strategies for identification, validation and prioritisation of combinations that lead to clinical trials. Finally, we make specific recommendations to the preclinical, small compound screening paradigm that could increase the likelihood of identifying tractable, combinatorial, small molecule inhibitors and better drug targets specific to GBM.

## Introduction

Glioblastoma remains a cancer of high unmet clinical need. Since 2005, the standard of care (SOC) treatment for younger, more physically fit patients has been surgery followed by radiotherapy and chemotherapy with TMZ. Unfortunately, surgical intervention is unable to prevent GBM progress due to the difficulty in obtaining the maximal resection of all tumour material whilst minimising surgical morbidity of essential brain regions. There is some evidence that the higher the level of tumor resection, aided by the development of image-guided surgical applications such as 5-aminolevulinic acid (5-ALA) or BLZ-100 to more readily define the tumour/brain tissue border, thus improving the amount of tumour surgically removed (1, 2), the better the resulting patient outcome (3). Coupled with surgical treatment is chemoradiation that combines six weeks of IR, delivering 60 Gy in 30 fractions to residual disease with daily TMZ (4), followed by six months of adjuvant TMZ using a 5-day over 28-day schedule (5). This standard therapeutic combination has not changed for almost 20 years but inevitably tumour recurrence occurs with a median survival for paitents treated with this schedule of under two years (6, 7).

Since the adoption of TMZ, there have been considerable efforts to broaden the number and range of therapeutics available to treat GBM (**Table 1**). However, the vast majority of systemic treatments, including all trials involving small molecules and biologics, have failed to improve patient outcomes. There are several factors prevalent in GBM driving this poor record of drug development and, consequently, the absence of improvement in patient outcomes; these include the striking range of molecular and cellular heterogeneity found in GBM tumours, the presence of both chemotherapeutic and radioresistant sub-populations of glioblastoma stem cells (GSC) in the tumour, the development and existence of protectant tumour microenvironments (TME) and of course, the existence of the blood-brain barrier which impedes the movement of small molecule and biologic therapeutics into brain tissue.

**Table 1:** Summary of Phase II-III Randomized Clinical Trials for Drug Combinations in GBM and their outcome.

Reference	Clinical Stage	# of patients	Clinical Benefit	Therapy	Disease state	Clinical trial identifier
Omuro et al., 2022 (8)	Phase III	560	Not met	Nivolumab + TMZ + RT *vs* TMZ + RT	Newly diagnosed GBM, unmethylated MGMT	NCT02617589
Lim et al., 2022 (9)	Phase III	716	Not met	Nivolumab + TMZ + RT *vs* TMZ + RT	Newly diagnosed GBM, methylated MGMT	NCT02667587
Sim et al., 2021 (10)	Phase II	125	Not met	RT+ Velaparib + TMZ *vs* RT + TMZ	Newly diagnosed GBM, unmethylated MGMT	ACTRN12615000407594
Nayak et al., 2021 (11)	Phase II	80	Not met	Pembrolizumab + Bevacizumab *vs* Pembrolizumab Alone	rGBM	NCT02337491
Peereboom et al., 2021 (12)	Phase II	47	Not met	RO4929097 (gamma secretase inhibitor)	rGBM	NCT01122901
Wen et al., 2021 (13)	Phase II	33	Positive	Dabrafenib + Trametinib	rGBM	NCT02034110
Cloughesy et al., 2020 (14)	Phase II/III	403	Not met	Vocimagene amiretrorepvec + flucytosine (retroviral replicating fector + prodrug) *vs* standard of care	Surgically resectable rGBM	NCT02414165
Natsume et al., 2020 (15)	Phase II	122	Not met	Interferonβ + TMZ *vs* TMZ alone	Newly diagnosed GBM	JCOG0911
Reardon et al., 2020 (16)	Phase II	73	Positive	BEV with either rindopepimut or a control injection of keyhole limpet hemocyanin	Relapsed EGFRvIII-expressing GBM	NCT01498328
Puduvalli et al., 2020 (17)	Phase II	90	Not met	BEV alone or with vorinostat	rGBM after RT with no prior BEV or HDAC inhibitors	NCT01266031
Lee et al., 2020 (18)	Phase II	115	Not met	BEV with and without trebananib	rGBM or gliosarcoma	NCT01609790
Cloughesy et al., 2020 (19)	Phase III	256	Not met	Ofranergene obadenovec (VB-111 viral therapy) + BEV *vs* BEV monotherapy	rGBM	NCT02511405
Rosenthal et al., 2020 (20)	Phase Ib/II	35	Not met	Buparlisib (pan PI3K inhibitor) + carboplatin or lomustine (compared with historical single agent data)	rGBM	NCT01934361
Galanis et al., 2019 (21)	Phase I/II	121	Not met	BEV + dasatinib or BEV + placebo	rGBM	NCT00892177
Cloughesy et al., 2019 (22)		35	Positive	Neo-adjuvant PD-1 blockade + surgery + adjuvant PD-1 blockade *vs* surgery + adjuvant PD-1 blockade	Surgically resectable rGBM	
Van den Bent et al., 2020 (23)	Phase II	260	Positive	Depatux-M (antibody-drug-conjugate) *vs* TMZ + Depatux-M	EGFR amplified rGBM	NCT02343406
Herrlinger et al., 2019 (24)	Phase III	141	Positive	TMZ *vs* CCNU-TMZ	Newly diagnosed GBM with methylated MGMT promoter	NCT01149109
Wakabayashi et al., 2018 (25)	Phase II	122	Not met	TMZ + interferonβ + RT *vs* TMZ + RT	Newly diagnosed GBM	JCOG0911
Wakabayashi et al., 2018 (26)	Phase II	170	Positive	BEV/IRI + RT *vs* TMZ + RT	Newly diagnosed, MGMT-nonmethylated GBM	NCT00967330
Chinnaiyan et al., 2018 (27)	Phase II	176	Not met	RT + TMZ + everolimus *vs* RT + TMZ	Newly diagnosed GBM	NCT01062399
Duerinck et al., 2018 (28)	Phase II	101	Not met	Axitinib monotherapy *vs* axitinib + CCNU	rGBM	NCT01562197
Wick et al., 2017 (29)	Phase III	437	Not met	CCNU + BEV *vs* CCNU alone	Progressive GBM	NCT01290939
Capper et al., 2017 (30)	Phase II	158	Not met	Galunisertib *vs* CCNU *vs* galunisertib + CCNU	rGBM	NCT01582269
Capper et al., 2017 (31)	Phase III	180	Not met	CIK cell immunotherapy + standard TMZ *vs* standard TMZ alone	Newly diagnosed GBM	NCT 00807027
Weller et al., 2017 (32)	Phase III	745	Not met	Rindopepimut + TMZ *vs* control injection of keyhole limpet hemocyanin	Newly diagnosed EGFRvIII expressing GBM	NCT01480479
Cloughesy et al., 2017 (33)	Phase II	129	Not met	Onartuzumab + BEV *vs* placebo + BEV	rGBM	NCT01632228
Gilbert et al., 2017 (34)	Phase II	117	Positive	BEV + IRI *vs* BEV + TMZ	rGBM	
Weathers et al., 2016 (35)	Phase II	71	Not met	Low dose BEV + CCNU *vs* standard dose BEV	rGBM	
Brandes et al., 2016a (36)	Phase II	91	Not met	RT/TMZ therapy and BEV or fotemustine	rGBM	NCT01474239
Brandes et al., 2016b (37)	Phase II	158	Not met	Galunisertib + CCNU or galunisertib monotherapy compared with CCNU + placebo	Relapsed or progressed GBM	NCT01582269
Brown et al., 2016 (38)	Phase II	38	Not met	Cediranib + gefitinib *vs* cediranib + placebo	First relapse/first progression of GBM following surgery+chemoradiotherapy	NCT01310855
Herrlinger et al., 2016 (39)	Phase II	182	Not met	BEV during RT followed by maintenance BEV + IRI or TMZ during RT followed by TMZ	Newly diagnosed GBM harbouring an unmethylated MGMT promoter	NCT00967330
Balana et al., 2016 (40)	Phase II	93	Not met	BEV + TMZ *vs* TMZ alone (neoadjuvant)	Unresected GBM	NCT01102595
Erdem-Eraslan et al., 2016 (41)	Phase II	114	Subtype benefits	CCNU, BEV or a combination of CCNU + BEV	First recurrence of GBM after TMZ + RT treatment	NCT01290939
Robins et al., 2016 (42)	Phase I/II	225	Not met	ABT-888 (velparib) in combination with TMZ	Recurrent TMZ resistant GBM	NCT01026493
Robins et al., 2016 (43)	Phase II	122	Not met	Carboplatin + BEV *vs* BEV monotherapy	rGBM	ACTRN12610000915055
Lee et al., 2016 (44)	Phase II	106	Not met	RT + TMZ with or without vandetanib	Newly diagnosed GBM or gliosarcoma	NCT00441142
(Blumenthal et al., 2015 (45)	Phase III	183	Not met	RT and O^6^-benzylguanine + BCNU *vs* RT and BCNU alone	Newly diagnosed GBM or gliosarcoma	NCT00017147
(Blumenthal et al., 2015 (46)	Phase II	138	Positive (HRQoL)	CCNU or BEV *vs* CCNU + BEV	rGBM	NCT01290939
(Blumenthal et al., 2015 (47)	Phase II	265	Failed	Standard and intensive cilengitide dose regimens in combination with TMZ/RT	Newly diagnosed GBM + unmethylated MGMT promoter	NCT00813943
Westphal et al., 2015 (47)	Phase III	149	Not met	Nimotuzumab + standard radiochemotherapy *vs* standard radiochemotherapy alone	Newly diagnosed GBM	NCT00753246
Schiff et al., 2015 (48)	Phase II	63	Not met	CT-322 with or without IRI	rGBM	NCT00562419
Penas-Prado et al., 2015 (49)	Phase II	155	Not met	Dose-dense TMZ in combination with isotretinoin, celecoxib, and/or thalidomide	Newly diagnosed GBM	NCT00112502
Penas-Prado et al., 2015 (50)	Phase II	25	Not met	Paclitaxel poliglumex (dose escalation) + TMZ + RT	Newly diagnosed GBM	NCT01402063
Stupp et al., 2014 (51)	Phase III	545	Not met	Cilengitide + TMZ/RT *vs* TMZ/RT alone	Newly diagnosed GBM + methylated MGMT promoter	NCT00689221
Taal et al., 2014 (52)	Phase II	140	Not met/uncertain	BEV alone *vs* CCNU alone *vs* BEV + CCNU	rGBM	NTR1929
Gilbert et al., 2014 (53)	Phase III	637	Not met	BEV + RT + TMZ *vs* placebo + RT + TMZ (BEV during RT, crossover allowed at progression)	Newly diagnosed GBM	NCT00884741
Hofland et al., 2014 (54)	Phase II	63	Not met	Neoadjuvant BEV + irinotecan *vs* BEV + TMZ (before, during and after RT)	Newly diagnosed GBM	NCT-00817284
Chauffert et al., 2014 (55)	Phase II	120	Not met	IRI + BEV as neo-adjuvant and adjuvant to TMZ-based chemoradiation *vs* TMZ-chemoradiation	Unresected GBM	NCT01022918
Batchelor et al., 2013 (56)	Phase III	325	Not met	Cediranib monotherapy *vs* cediranib + CCNU *vs* CCNU + placebo	rGBM	NCT00777153
Stragliotto et al., 2013 (57)	Phase I/II	42	Not met	Valganciclovir *vs* placebo (in addition to standard therapy)	rGBM	NCT00400322
Stragliotto et al., 2013 (58)	Phase III	250	Not met	Intraoperative perilesional injection of sitimagene ceradenovec followed by ganciclovir in addition to standard care or resection and standard care alone	Newly diagnosed GBM amenable to complete resection	2004-000464-28
Shibui et al., 2013 (59)	Phase II/III	111	Not met	Nimustine hydrochloride + procarbazine + RT *vs* procarbazine alone + RT	Newly diagnosed anaplastic astrocytoma (n=30) and GBM (n=81)	UMIN-CTR C000000108
Nabors et al., 2012 (60)	Phase II	112	Positive	Cilengitide (either 500 mg or 2000 mg) + standard RT/TMZ	Newly diagnosed GBM	EORTC 26981
Nabors et al., 2012 (61)	Phase II	68	Positive	BEV and everolimus + standard RT/TMZ	Newly diagnosed GBM	
Kim et al., 2011 (62)	Phase III	168	Not met	RT followed by adjuvant TMZ with or without neoadjuvant nimustine-cisplatin chemotherapy	Newly diagnosed GBM	
Dresemann et al., 2010 (63)	Phase III	240	Not met	Imatinib and hydroxyurea *vs* hydroxyurea monotherapy	rGBM after resection, RT and first-line chemotherapy (preferably TMZ)	NCT00154375
Friedman et al., 2009 (64)	Phase II	167	Positive	BEV alone and in combination with IRI with or without concomitant enzyme-inducing antiepileptic drugs	rGBM	NCT00345163
Buckner et al., 2006 (65)	Phase III	401	Not met	Carmustine + cisplatin compared with carmustine alone + standard RT or accelerated RT	Newly diagnosed GBM	
Sotelo, Briceño & López-González, 2006 (66)	Phase III	30	Small sample size	Adding chloroquine to conventional therapy	Histologically confirmed GBM in first or second recurrence or relapse	NCT00224978
Stupp et al., 2005 (7)	Phase III	573	Positive	RT + TMZ	Newly diagnosed GBM	NCT

BEV, bevacizumab; RT, radiotherapy, TMZ, temozolomide; IRI, irinotecan; BCNU, 1,3-bis(2-chloroethyl)-1-nitrosourea; PCV, procarbazine, lomustine, and vincristine; CCNU, lomustine; ACNU, 1-(4-amino-2-methyl-5-pyrimidinyl)methyl-3- (2-chloroethyl)-3-nitrosourea hydrochloride; 5-FU, 5-fluorouracil; VM-26, teniposide; PCNU, nitrosourea analogue; CT-322, VEGFR2 antagonist; DTIC, dacarbazine; rGBM, recurrent glioblastoma.

In this article, we summarize the current state of clinical trials in GBM and highlight the fact that poor clinical translation of potential GBM therapeutics could, in part, be attributed to the failure of drug development campaigns to incorporate many of the key pathophysiological features and sources of heterogeneity of human GBM into the earliest stages of the drug development pipeline. We review the steps academic research scientists are taking to address these failures and argue that high throughput screening to identify novel GBM combinatorial targets for drug development must incorporate the use of a diverse set of patient-derived GSC lines from as wide a cross-section of the GBM disease spectrum as possible, should include chemotherapeutic and radio-resistant cells, and be screened in physiologically relevant conditions including hypoxia in combination with radiotherapy +/- TMZ. Next, we discuss the rationale for current GBM combination trials is not always clear and we wish to emphasise the need for more systematic and transparent strategies for identification, validation and prioritization of combinations that lead to clinical trials. Finally, we make some specific recommendations to the preclinical, small compound screening paradigm that we feel could increase the likelihood of identifying tractable, combinatorial, small molecule inhibitors and better drug targets specific to GBM.

## GBM intra/inter heterogeneity as a driver of resistance

**Figure 1:**
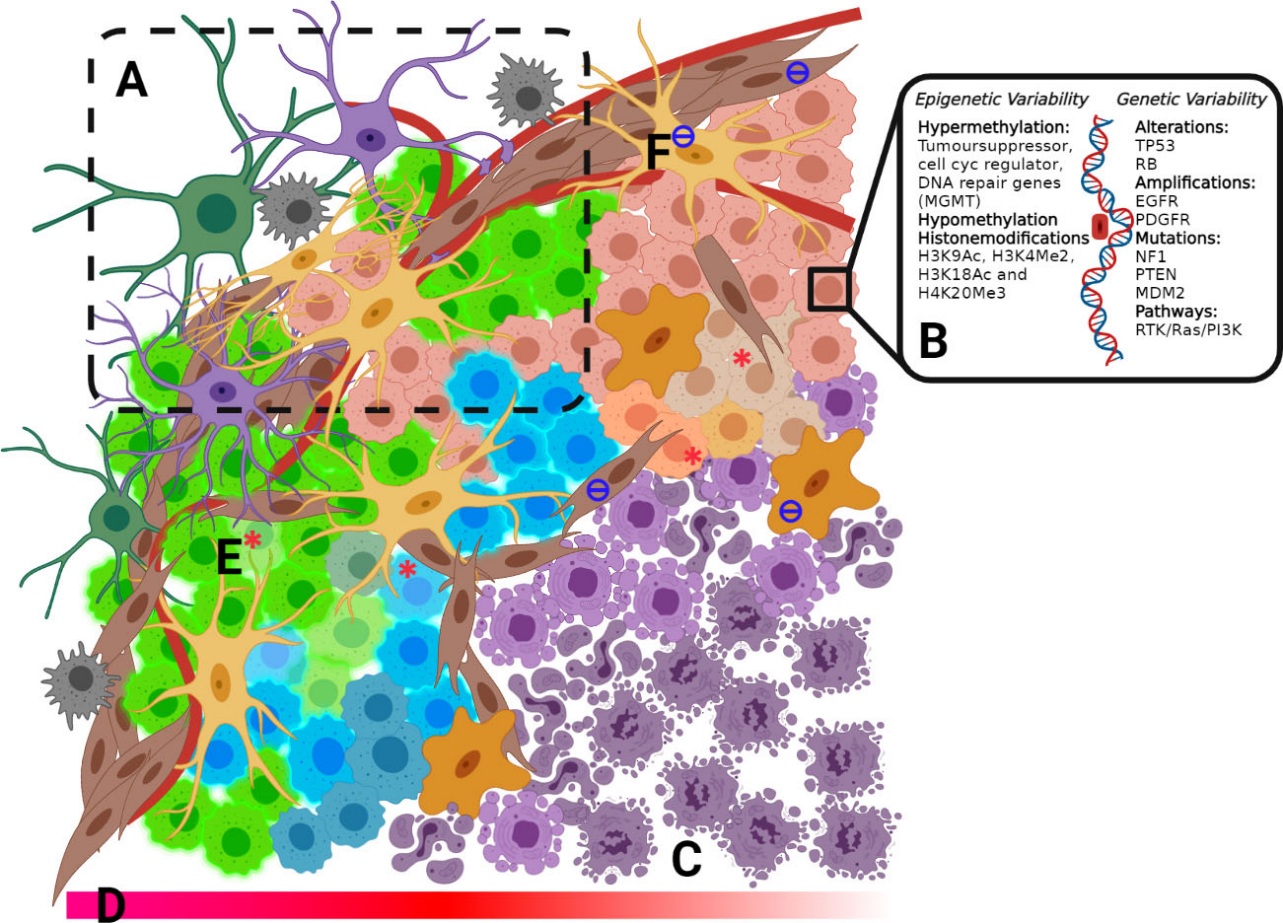
**(A)** Invasive Zone includes a mixture of cell types, including fibroblasts, oligodendrocytes, macrophages, microglia, astrocytes and GBM tumour cells. **(B)** Epigenetic and genetic variability **(C)** Necrotic core **(D)** Decreasing O_2_ concentration into the tumour core **(E)** GSC from each of the 4-subtypes: Classical, pro-Neura, Neural and Mesenchimal within microenvironmental niches **(F)** Tumour-associated fibroblast and macrophages.

The degree of intra-/inter-heterogeneity of GBM tumor profiles is particularly extensive (**Figure 1**) and has proven to be an important factor in determining long term cancer survival (67). Whole-genome sequencing of tumours has revealed extensive molecular heterogeneity within (68) and between (69, 70) patients, presenting substantial challenges to the identification and prediction of targeted (precision) therapeutic strategies. Tumours exhibit many somatic mutations in both coding (69) and non-coding regions (71) affecting both gene copy number and coding mutations, as well as regulatory elements for specific genes. However, clearly not all are driver mutations. Instead, the majority are ‘passenger’ mutations that may confer additional survival advantages under the selective pressures of drug or radiation treatment and promote clonal evolution during therapy, providing opportunities for drug resistance to occur (67).

Tumour heterogeneity and the implications of mutations on GBM pathology are seen at the genomic level when the WHO re-classification of GBM based on the mutational status of isocitrate dehydrogenase (IDH) (72) and recently completed a further re-classification to include one additional feature such as of loss chr 10 or gain chr 7, EGFR ampli, or TERT (73). IDH mutant status in GBM has a significant impact on prognosis. Although IDH-mutated glioma generally exhibit a better disease outcome (74), the incidence of IDH mutations in secondary tumour suggests that lower-grade glioma with IDH mutation often recur after having undergone malignant transformation to a higher grade. In addition, IDH-mutated glioma is more likely to develop a hypermutation phenotype (75). Ongoing research has generated a growing list of molecular and protein differences found in GBM tumours (**Figure 1**) relating to treatment response and patient outcome (76). These mutations have been well catalogued with strong correlation across testing platforms (69, 77, 78).

An example of tumour heterogeneity at the epigenetic level occurs with the methylation status of O6-methylguanine-DNA methyltransferase (MGMT). When MGMT is silenced through methylation, it is unable to repair the DNA damage induced by TMZ (79) which has been associated with improved survival. Protein arginine methyltransferase 5 (PRMT5) gene expression in GBM is inversely correlated with survival (80). Recent studies show that pharmacological inhibition of PRMT5 suppresses the growth of GSC cultures and significantly prolongs the survival of mice with orthotopic patient-derived GBM xenografts (81). These emerging studies, together with observations that mutations in genes linked to chromatin organisation, are a common feature of GBM (69), implying that epigenetic processes may be a common driver of GBM across heterogeneous subtypes.

Another confounding factor in the resistance of GBM to chemotherapeutics is the developmental state of GBM cells within the tumour. GBM hijacks mechanisms of neural development to produce subcompartments of GSCs that exhibit resistance to radiotherapy and chemotherapies contributing to tumour-propagating potential and recurrence following treatment (82–85). In a recent study, an integrative approach incorporating single-cell RNA-sequencing, bulk genetic and expression analysis of multiple patient tumours, functional assays and single-cell lineage tracing was employed to derive a unified model of cellular states and genetic diversity in GBM (86). The authors postulate that malignant cells in GBM exist in four main cellular states that recapitulate distinct neural cell type features; Astrocyte like, Oligodendrocyte precursor cells, Neural progenitor cells, and Mesenchymal like.

Along with genetic/epigenetic inputs and GSC, the TME drives cellular heterogeneity and subsequently treatment outcomes in GBM. A major structural component of the TME is established by a complex mixture of >15 glycosylated extracellular matrix (ECM) proteins, which establishes a physical and biochemical niche which impacts the interactions between tumour cells and the ECM, including regulation of cell fate, differentiation and migration (87) and are critical to the invasiveness and malignancy of GBM. Importantly, the TME in GBM helps to create avascular regions within the tumour, which leads to decreased tumour O_2_ tension and the development of a necrotic core in the tumour, a hallmark of GBM. These hypoxic conditions play a crucial role in protection against chemotherapy and radiation, likely through low O_2_ content in the tumour as radiation treatment relies on O_2_ and upregulation of numerous proliferative and metastatic pathways attributed to the hypoxia-inducible factors (HIF1α and HIF2α) (88). HIF has been implicated in the dedifferentiation of GBM cells driving the proneural-to-mesenchymal transition, believed to be a hallmark of resistance in recurrent tumours. Another important characteristic of the GBM microenvironment is the significant infiltration of resident microglia and peripheral macrophages. Recent research has implicated these tumour-associated microglia and macrophages in central aspects of tumour development in GBM, including proliferation, angiogenesis and immunosuppression (89), which contributes to the chemoradiation resistance and rapid progression of the disease.

## Glioblastomas stem cells and the rationale for combinations

The need for a combination strategy can be rationalized by the well-established cellular heterogeneity of brain tumours. There is evidence to suggest that brain tumours may arise from a stem cell origin (90). The proportion of stem cells in tumours, and the proteins they express (such as CD133 and Ki67), often determine the aggressiveness of recurrent GBM and can inversely correlate with patient outcomes (91, 92). Additionally, from a collection of experimental approaches (68, 86, 93), this stem cell origin gives rise to a proliferative hierarchy in primary GBM, leading to the presence of a range of cell states, from stem cell to differentiated cells within a tumour. Developmental and lineage subtypes invariably generate therapy-resistant populations due to higher cellular entropy (94), providing various genetic and epigenetic mechanisms stem cells use to evade death from radiation and chemotherapy treatment (95, 96).

**Figure 2:**
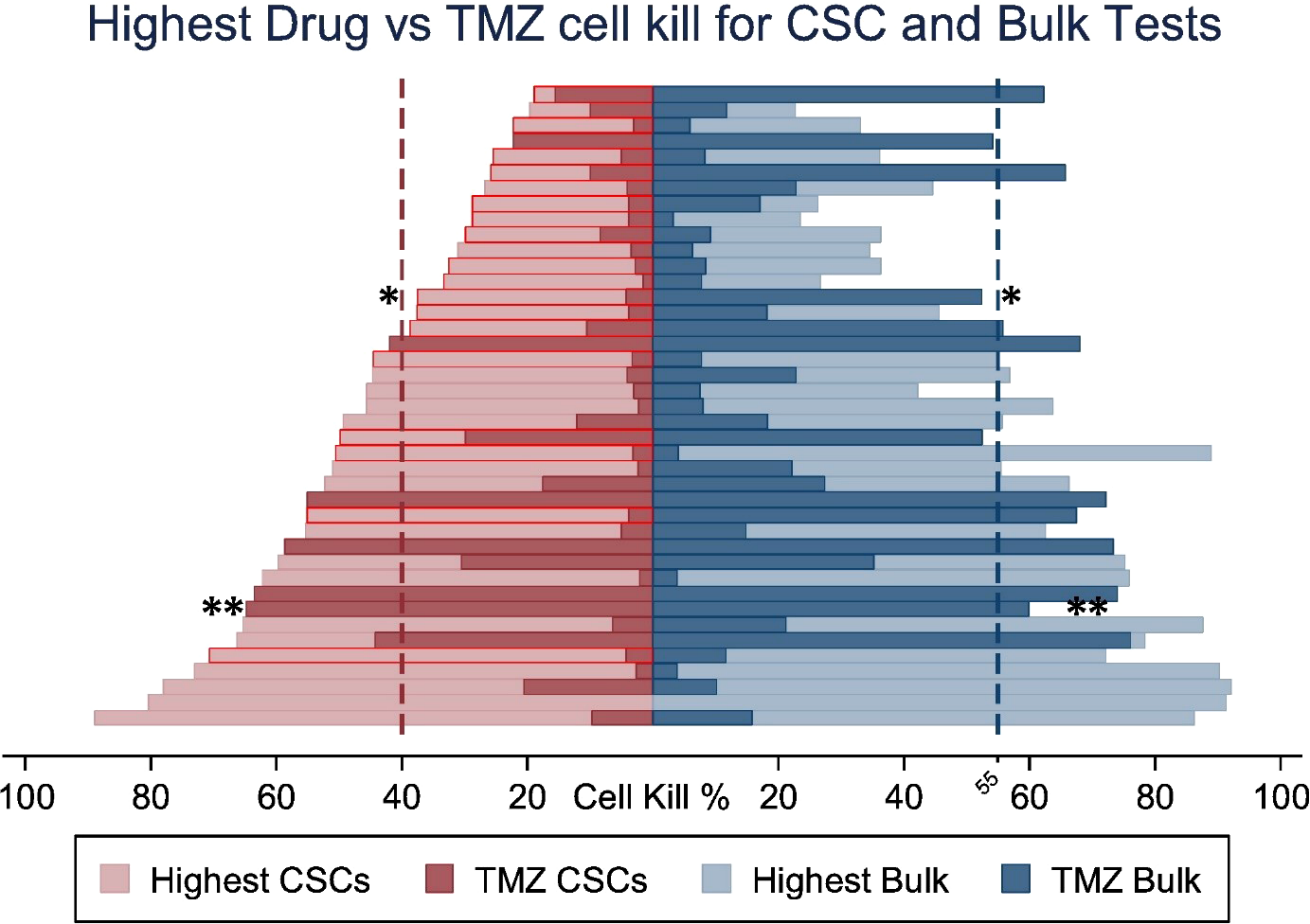
Percent cell death for the most cytotoxic drug and TMZ for each patient comparing Cancer Stem cell (CSC) *vs* bulk of tumour cell sensitivity to TMZ. Optimal therapies with highest cell death shown in light colours and cell detah by TMZ in dark. CSC results outlined in red show patients whose optimal therapy differs from the optimal therapy identified by the bulk of tumour cells test * p <0.05, ** p < 0.01. Adapted from Howard C.M., et al., 2017 (97).

Stem cells are increasingly becoming the target cell for further research into understanding GBM and treatment responses. The variable responses of tumour cell populations to standard GBM treatment implies that it is sensible to consider the combination of more than one drug; one of these may target stem cells and the other target non-stem cells in the bulk tumour. Data increasingly show the importance of targeting both subpopulations. For example, using patient-derived cells from biopsies, it has been reported that for greatest patient benefit, both stem cells and ‘tumour bulk’ cells need to be killed by >40% and >55%, respectively (97). Additional evidence that stem cells and bulk cell populations respond differently to drugs has been gathered from the development of a chemotherapeutics assay termed ChemoID (97). The assay involves culturing GBM stem cells and tumour bulk cells from patient biopsy material and assessing the *in vitro* killing ability of a panel of chemotherapeutic drugs. Significant differences in sensitivity to TMZ between stem cells and bulk cells within the same patient tumour sample (**Figure 2**) have been observed.

In the majority of samples tested, bulk cells were more sensitive to TMZ than stem cells; thus, TMZ-treatment will not likely have significantly killed the stem cell population. Although not the ideal chemotherapeutic drug, due to insensitivity in a large proportion of patients, TMZ may be of greater value for use against bulk tumour cells, opening up the possibility of using TMZ combined with additional drugs to attack the heterogeneous tumour cell complement within the tumour mass.

## Hypothesis-driven drug additions to the standard-of-care regimen of TMZ and IR

The treatment regimen of surgery followed by TMZ and IR can slow the progression of GBM tumours, but it is not generally curative. The simplest solution to increasing the effectiveness of treatment would be to incorporate an additional therapeutic agent into the SOC treatment regimen to target one or more specific GBM tumour cell survival pathways to yield a combinatorial or synergistic effect. An example of a hypothesis-driven combinatorial approach has been to target tumour angiogenesis, increased tumour vascularisation supports GBM growth and survival in the face of radiochemotherapy. Vascular Endothelial Growth Factor (VEGF) is over-expressed in a variety of metastatic tumours and bevacizumab (Avastin), an anti-VEGF antibody, was first approved for treatment in metastatic colorectal cancer in 2004 (98) and later for GBM treatment in 2009 (6). Trials in in newly diagnosed GBM, such as the Avaglio trial or RTOG 0825 trial (53, 99) showed no increase in overall survival although progression-free survival times were extended by 3-4 months with bevacizumab (53, 100). To date, it remains the only approved (not in EU) antibody treatment for recurrent GBM (101). The difficulty in obtaining significant clinical benefits for anti-angiogenic therapies in newly diagnosed GBM has been summarized in retrospective meta-analyses reviews (102, 103).

Another promising approach that has had significant success in many advanced malignancies is the use of immune checkpoint inhibitors (ICI) targeting key checkpoint pathways including the cytotoxic T-lymphocyte-associated antigen 4 (CTLA-4)/B7 and programmed death 1 (PD-1)/programmed cell death ligand 1 (PD-L1), reviewed by Zhang et al., 2021 (104). Despite their successes, ICIs are yet to improve survival in GBM. By addressing the restrictive nature of the TME, the timing of checkpoint inhibition and employing novel combinatorial strategies of ICIs this strategy could still provide effective therapeutic options in the future for GBM (105).

Additional small molecules have been tested as potential treatments based on GBM driver pathways. A recent review listed 90 clinical trials with 85 different compounds either as monotherapy or in combination with standard treatment, the majority are tyrosine kinase inhibitors (6), and alternative alkylating agents (Clinicaltrials.gov). Unfortunately, so far, none of these has delivered a beneficial treatment (**Table 1**).

Data and clinical trials from hypothesis-driven multiple drug combination therapies are less well documented than those seen for single or double additions to SOC treatment. Here, the strategy is to target multiple molecules or pathways implicated in tumour development and survival. One such hypothesis is the Co-ordinated Undermining of Survival Pathways (CUSP9) in GBM, which utilizes nine clinically approved growth-factor inhibiting drugs alongside TMZ treatment for recurrent GBM. (now called CUSP9 with slightly altered panel composition - **Table 2**) (131).

**Table 2:** List of CUSP9 drugs and their expected benefit in treating GBM. Adapted from Kast R.E., 2013 (106).

DRUG	EXPECTED BENEFIT	REF.
aprepitant	Nausea reduction, inhibit growth by blocking NK-IR.	(107, 108)
artesunate	Increases ROS, empirical anti-glioma effects, survivin inhibition	(109, 110)
sertraline	Empirical longer OS, improved mood, documented anti-proliferation effects in glioma cells.	(111–113)
captopril	Empirical longer OS, MMP-2 & MMP-9 inhibition, prevents AT-2 stimulation, lowers IL-18, stimulated VEGF, TNF & IL-8.	(114–117)
auranofin	Thioredoxin reductase inhibition, cathepsin B inhibition i.e. ROS, empirical [& potentially dangerous] synergy with artesunate.	(118, 119)
nelfinavir	HSP90 inhibition, MMP-2 & MMP-9 inhibition, decreased signaling at multiple receptors, i.e. TGF-β, increased ROS, decreased AKT activation, lower VEGF, IL-8, ICE inhibition.	(120–122)
temozolomide	A common and accepted treatment for recurrent GBM	(79)
disulfiram	ALDH inhibition, glutathione inhibition, increases ROS, lowers IL-18, stimulated VEGF, TNF, & IL-8, MMP-2 & MMP-9 inhibition, proteasome inhibition, SOD inhibition, P-glycoprotein inactivation, MGMT inhibition.	(123–128)
Cu gluconate	Adequate Cu may be a requirement for disulfiram activity.	(129)
ketoconazole	Drug efflux inhibitor at BBB, permits higher brain ritonavir (or nelfinavir) concentrations, 5-lipoxygenase inhibitor, thromboxane synthase inhibitor, empirical anti-glioma effect.	(121, 130)

**Figure 3:**
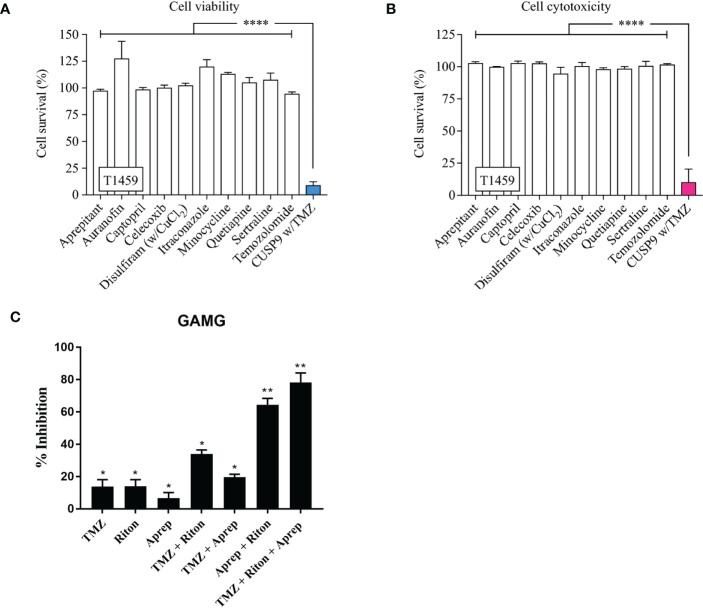
CUSP9 with TMZ. **(A, B)** Individually, the drugs in the CUSP9 or TMZ did not reduce cell survival, evaluated by the cell viability or cytotoxicity assay; a significant effect was observed when applied as a drug combination in CPCs (**** *p* < 0.0001, one-way ANOVA). **(C)** Percentage of growth inhibition of human GAMG cells following the addition of temozolomide (TMZ), ritonavir (Riton) and aprepitant (Aprep) in monotherapy; temozolomide + ritonavir, temozolomide + aprepitant and aprepitant + ritonavir in combination therapy (* p <0.05, ** p < 0.01). Adapted from Skaga E., et al., 2019 (132) and Kast R.E., et al., 2016 (105).

A recent study in patient-derived GSC based on the CUSP9 drug panel, highlights individually these drugs have little effect, but in combination the tumour killing effect can become significant (**Figures 3A, B**) (132).

**Figure 3**: CUSP9 with TMZ. **A**, **B** Individually, the drugs in the CUSP9 or TMZ did not reduce cell survival, evaluated by the cell viability or cytotoxicity assay; a significant effect was observed when applied as a drug combination in CPCs (both *p* < 0.0001, one-way ANOVA). **C** Percentage of growth inhibition of human GAMG cells following the addition of temozolomide (TMZ), ritonavir (RITON) and aprepitant (APREP) in monotherapy; temozolomide + ritonavir, temozolomide + aprepitant and aprepitant + ritonavir in combination therapy. Adapted from Kast RE,. et al., 2015 (131) and Skaga E., et al., 2019 (132)

A further study highlights two of the CUSP9 drugs, aprepitant and ritonavir (133), tumour cell killing is either poor (aprepitant 6%) or equivalent to TMZ alone (ritonavir 14%). However, when used in combination, aprepitant and ritonavir show significantly increased synergistic inhibition which was increased when TMZ was also added for a triple combination (**Figure 3**). The CUSP idea and initial promising results using tumour cell lines imply that successful combination therapies for GBM may well require more than two or even three drugs.

## Non-hypothesis driven drug additions to the SOC regiment of TMZ and IR

With the failure of clinical trials resulting from hypothesis-driven drug combination studies, non-hypothesis driven, high throughput screening (HTS) strategies are now coming to the forefront of academic and pharmaceutical industry research (**Table 3**).

**Table 3:** Non-hypothesis driven HTS campaigns to identify novel targets, drug-combinations or small compounds for the treatment of GBM.

Reference	Cell type	Assay format	Assay endpoint	Screen size	No. of hits
Skaga et al., 2019 (134)	GBM biopsies from 12 patients were used to establish cell cultures.	Cells were plated at 5000 cells/well in a 96-well plate under sphere conditions, cultured for 24 h before the addition of drugs and further incubated for 72 h.	Viability was assessed using Cell Proliferation Kit II XTT (Roche) solution incubated for 24 h before analysis.	The oncology drug collection consisted of 461 FDA/EMA-approved anti-cancer drugs and investigational compounds with a broad range of molecular targets.	All drugs have (i) been tested in clinical trials of GBM (nintedanib, paclitaxel, topotecan), (ii) are currently in clinical trials of GBM (belinostat (NCT02137759), sapanisertib and selinexor (clinicaltrials.gov) or (iii) represent drugs within a class that are being investigated in GBM (carfilzomib; proteasome inhibitors, idasanutlin; mdm2 inhibitors, clinicaltrials.gov).
Lucki et al., 2019 (135)	Patient-derived GBM CSC cultures	uHTS Luciferase-based survival assay (~10^6^ molecules) followed by high content imaging-based selective toxicity assay (~8000 molecules) then a caspase 3/7 activation assay (~200 molecules)	Induction of apoptosis	∼10^6^ small molecules	Identified a small molecule, RIPGBM, that selectively induces apoptosis in GBM CSCs *in vitro* and significantly decreases tumour size *in vivo* in a physiologically relevant, patient-derived intracranial xenograft mouse model.
Wilson et al., 2019 (136)	Two spheroid cell lines, JHH-136 and JHH-520,	High-throughput drug screen using an 11-point dose response. Drug combination screening of 30 compounds (435 combinations) using 5 concentrations of one and 5 concentrations of a second compound. Follow up drug screen of 46 drug combinations.	Cell viability or apoptosis	The Mechanism Interrogation PlatE (MIPE) 4.0 is a collection of 1912 small molecules that target signalling pathway components that are altered in many different cancers.	Drug mechanisms that were cytotoxic in both cell lines were Hsp90 and proteasome inhibitors. JHH-136 was uniquely sensitive to topoisomerase 1 inhibitors, while JHH-520 was uniquely sensitive to Mek inhibitors. Drug combination screening revealed that PI3 kinase inhibitors (GDC-0941) combined with Mek (PD0325901) or proteasome inhibitors (marizomib) were synergistic. *In vivo* revealed that Mek inhibition alone was superior to the combination treatments.
Quereda et al., 2018 (137)	Patient-Derived Glioma Stem Cells	1536-well spheroid-based proliferation assay using 3300 approved drugs.	3-D Cell proliferation was assessed after another 72-hour incubation using CellTiter Glo reagent	A collection of 3,291 clinically approved drugs assembled at the Scripps Research Institute Molecular Screening Center (SRIMSC).	Bortezomib was observed to be more potent against GBM6 GSCs (GBM6 spheroids or in laminin) than against the differentiated GBM6 (bulk tumour) or the U87 cell line, suggesting Bortezomib may specifically affect the master regulators of the GSCs.
Yu et al., 2018 (138)	7 patient derived cancer stem cell lines: 5 GBM: 1 Gliosarcoma: 1 Gliomatosis cerebri	High content screening assay, two 384-well plate formats performed in tandem in serum-free conditions: 1. Adherent monolayer on laminin-coated plates. 2. 3D neurospheres Each compound screened as a 5-point dose-response	HCI DRAQ7 and Hoechst 33342 to identify compounds for cell death and proliferation. Triage in 3D neurospheres. *In vivo* validation of hits in orthotopic model following stereotactic injection of cells into the right striatum of NOD-SCID Gamma Null (NSG) mice.	83 chemotherapy drugs with and without irradiation	Drug hits demonstrating significant reduction of tumour growth *in vivo* include: Mitoantrone; Bortezomib, Actinomycin D and Paclitaxel. Only paclitaxel (in a form that was linked to a biodegradable, water-soluble polyglutamate polymer) was evaluated in a phase II trial Paclitaxel poliglumex (dose escalation) + TMZ + RT Newly diagnosed GBM (NCT01402063).
Lee et al., 2017 (139)	NHA-astrocyte AGM and four patient-derived GBM cells from four GBM patients	3D cell-based high-throughput screening method reflecting the microenvironments using a micropillar and microwell chip platform	Cell viability or a high-dose heat map of the cytotoxicity and efficacy of 70 compounds	70 compounds	Among the 70 compounds tested, cediranib (a potent inhibitor of vascular endothelial growth factor (VEGF) receptor tyrosine kinases) exhibited the lowest cytotoxicity to astrocytes and high efficacy to GBM cells in a high-dose heat map model.
Lun et al., 2016 (140)	13 independent genetically distinct patient-derived brain tumour-initiating cell lines	HTS was performed using 96-well microplates and two drug concentrations. Secondary validation was performed on a subset of BTICs using 8-point, 3-fold serial dilutions of compounds.	72h Alamar blue – proliferation assay	NIH clinical drug library that contains 446 compounds that have been used in human clinical trials, and a ToolKit library (OICR, Canada) containing 160 compounds	Montelukast, clioquinol & disulfiram. Disulfiram, an off-patent drug previously used to treat alcoholism, in the presence of a copper supplement, showed low nanomolar efficacy (including those resistant to TMZ and the highly infiltrative quiescent stem-like population). Validated *in vivo*, prolonged survival in patient-derived BTIC models established from both newly diagnosed and recurrent tumours.
Denicolaï et al., 2014 (141)	GBM6 and GBM9 stem-like cell lines and on U87-MG and U251-MG cell lines	Scratch assay and flash-cytometer acquisitions (drug treatment for 3 days)	Inhibitory efficacy on cell migration and proliferation	Prestwick chemical library^®^ of 1120 molecules (all approved drugs)	Proscillaridin A, a cardiac glycoside inhibitor of the Na+/K+ ATPase pump. Also selected: emetine dihydrochloride and strophantidin.
Hothi et al., 2012 (142)	Glioma stem cell cultures were established from freshly resected tumour tissues	HTS was performed on five patient-derived cultures (384-well plates, 8-point dose-response curves). Further ToxCount cell viability assays, proteasomal chymotrypsin-like activity assays, aldefluor analysis and flow cytometry.	CellTiter-Glo was added to individual wells and, following 20 minutes incubation on an orbital shaker, luminescence was measured, and percentage cell viability was calculated.	Chemical library of 2,000 compounds.	Identified disulfiram (tetraethylthiuram disulfide, Antabuse^®^, DSF), a clinically approved drug for the treatment of alcoholism, as a potent inhibitor of multiple patient-derived GSCs.
Wang et al., 2012 (143)	Human GBM cell lines U87MG and U251MG	An HTS assay for cell growth and invasion followed by traditional cell growth and invasion assays.	Cell growth and invasion.	LOPAC1280 pharmacologically active compounds that influence most cellular processes and cover all major drug target classes.	Ten validated active compounds were obtained, of which six have been previously reported and four newly identified compounds 6-nitroso- 1,2-benzopyrone, S-(p-azidophenacyl) glutathione, phenoxybenzamine hydrochloride and SCH-28080
Visnyei et al., 2011 (144)	Patient-derived GBM stem cells	Multiple screening strategies. The HTS was done in a 384- well plate format at concentrations recommended by the manufacturers	(1) Cell viability GSC derived from one tumour. (2) differential effect between any of the two samples (3) repeat (2) on a panel of serum- and sphere-derived GBM samples (4) evaluate for their GSC selectivity and gene-target specific qRT-PCR–based screens to determine their inhibitory effect on key GBM regulator genes.	31,624 small molecules from 7 chemical libraries.	Highest priority compounds validated *in vivo*. Compounds #5560509, #5256360, OLDA, and emetine inhibited tumour formation in immunosuppressed animals.

Traditionally in HTS, very large collections of small compound libraries with diverse chemical structures have been employed by the pharmaceutical industry to identify starting compounds for drug development programs. However, this process is generally less successful at the academic level due to the prohibitive infrastructural costs required to develop and produce larger screens, as well as the cost to develop a small compound hit into a clinical drug candidate. As a result, many academic screening facilities have instead focussed on developing more physiologically relevant phenotypic assays which better recapitulate key areas of disease biology (145) and then screen smaller, focussed libraries such as “drug repurposing” (146–148), probes for target discovery (149–151) or pharmacologically active small compound sets (152). This focussed approach has the advantage of providing a better understanding of the molecular basis of the disease while simultaneously providing the opportunity to exploit existing therapeutics and compounds with known safety profiles, small compounds that possess drug-like properties and compounds with known protein targets, and can provide a more direct path to clinical translation (153).

Academic drug screens have proven to be successful at identifying novel repurposing opportunities in a variety of other cancers (154), so are good candidates as potential additions for GBM therapy (137, 155, 156). While a number of HTS and drug repurposing screens have been performed recently in GBM, with several hits being tested in early-phase clinical trials (**Table 3**), such screening hits have yet to translate into robust randomized phase II/III testing (**Table 1**).

Screens using smaller but diverse small molecule libraries are also becoming more commonplace, including uncharacterized molecules that could lead to a novel therapeutic discovery program rather than just a repurposing opportunity. One example study using approximately 31,000 small molecules identified 8 compounds showing greater inhibitory effect in GSC cultures compared with non-stem cell-enriched GBM cultures (144). In addition, another two compounds inhibited four GBM hub genes ASPM, MELK, FOXM1b and TOP2a. From these xenotransplants of GSC enriched tumour cells pre-treated with four candidate compounds (Emetine, #5560509, #5256360 or OLDA) displayed significantly reduced tumour mass volume suggesting a targeted effect on the tumour initiating stem cells. However, no further studies have been done on these compounds.

For larger cancer focussed HTS employing pharmacologically active compound libraries (157) or collections of probe libraries targeting, for example, kinases (158) or epigenetic modifiers (159), the size of some of the available drug libraries is in the hundreds/thousands, resulting in an exponentially increasing the number of possible drug combinations. Therefore, screening all possible pairwise or higher-order combinations would be a huge task. For time and financial reasons, combinatorial drug screening will, therefore, realistically need to be constrained. If it is likely that one or more drugs would be required in addition to TMZ, what are the criteria that would identify a sole molecule as advantageous to use in combination? This may be an important consideration in the light of, for example, the CUSP9 data showing little activity as sole agents but significant effect in combination. The simplest and quickest screening format would be to identify candidates that already show a significant amount of tumour killing ability by themselves, and the studies described above show that potential drugs can be selected. In contrast, however, that format would not have selected any of the CUSP9 drugs which look potentially promising.

## Non-hypothesis (screening) combinatorial studies from CRISPR screens

The development of clustered, regularly interspaced short palindromic repeats (CRISPR)/Cas gene-editing technologies can be deployed in the earliest stages of drug development to enable unbiased identification of new molecular targets through genome-wide CRISPR screens in cells or small model organism-based models of disease (160–162). A study using genome-wide pooled CRISPR screening across a panel of patient-derived GCS cultures was recently reported to define the molecular determinants governing GSC growth and survival (163). The screen was performed in the presence of a lethal dose of TMZ to identify genes modulating TMZ sensitivity revealing mechanisms of therapeutic resistance and new strategies for combinatorial therapy, including GSC self-renewal, GSC stemness and proliferation, as well as regulators of Cell Stress Response Pathways which all appear essential for GSC viability and fitness. Positive selection screens performed in two patient-derived GBM cell cultures treated with a lethal dose of TMZ revealed core members of the mismatch repair (MMR) pathway. To identify mechanisms of intrinsic resistance of the GSC population to TMZ, the authors performed negative selection screens in the presence of a sublethal dose of TMZ. These studies identified the Fanconi anemia pathway’ (163)

Other CRISPR-Cas9 screenings have identified further potential targets that sensitize GBM cells to TMZ, including suppressing the NF-κB/E2F6 signalling axis (164). In another study, the authors applied a pooled CRISPR-Cas9 screening approach to the U138 human glioma line in a transwell invasion assay (165). Isolation and sequencing of cells that display accelerated invasion through the transwell filter identified inhibition of mitogen-activated protein kinase 4 (MAP4K4) as a potential therapeutic target for GBM invasion (165). A further study used CRISPR interference (CRISPRi) to screen 5689 Long non-coding RNAs (lncRNAs) loci in human GBM cells, identified 467 hits that modify cell growth in the presence of clinically relevant doses of fractionated radiation and 33 of these lncRNA hits sensitize GBM cells to radiation (166). Follow up studies demonstrated that antisense oligonucleotides targeting lncGRS-1 selectively decrease tumour growth and sensitize glioma cells to radiation therapy (166). The evolution of CRISPR/Cas 9 screening, including arrayed screening strategies across more definitive GBM phenotypic assay endpoints and pooled screens to identify sensitizers of additional drug classes are well placed to identify further therapeutic targets and drug combination hypotheses in GBM.

## The role of hypoxia, tumor-microenvironment, and non-neoplastic cells for IR and drug resistance in GSC

Tumour heterogeneity and the presence of GSC alone does not explain the lack of successful clinical translation of promising preclinical discoveries in GBM (**Table 1**) and may in fact be due to experimental reasons; chief among them is the growing realisation that currently, the *in vitro* and *in vivo* models of GBM used in preclinical drug discovery do not completely recapitulate tumour biology within patients. GBM cells and their progenitor GSC dynamically respond to their local microenvironment through a multitude of bidirectional communications resulting in the permissive conditions for tumour growth and invasion. *In vitro* models employed to date have sought to encompass, to varying degrees, some of this environmental complexity in the high throughput setting.

In initial experiments isolating and characterising GSCs, a neurosphere culture paradigm was used successfully with GSCs (167, 168). Further advances have seen the field introduce adherent culture grown on different base layer coatings of ECM components, including collagen and laminin. These methods, previously established for fetal and human neural stem cells, overcame limitations of neurospheres, whilst generating pure and expandable populations of GSCs, are (169) readily amenable to an HTS format and have been extensively used in drug discovery for GBM since their establishment (144, 170).

A major hallmark of GBM and a vital component of the TME that has not featured in drug discovery efforts to date is the hypoxic core of tumours. These regions result from increased cell proliferation overcoming oxygen supply (171), in turn generating an environment that supports tumour progression through GSC maintenance, proliferation, and drug resistance (172–174). In current GBM research the majority of *in vitro* cell models do not account for the effects of the hypoxic TME with cells cultured in atmospheric oxygen with a partial oxygen pressure of 159 mmHg. Studies quantitating pO_2_ in gliomas recorded a highly significant number of pO_2_ readings in patients with GBM less than 2.5 mmHg (175, 176), levels known to reduce the efficacy of radiotherapy where cellular sensitivity is halved when pO_2_ is below 3 mmHg (177). Recent research generated a novel 3D *in vitro* model with common GBM cell lines, U87, U251, and SNB19, to study hypoxic effects in the context of TMZ resistance (178), highlighting the significant role of hypoxia on drug resistance.

The role of non-neoplastic cells in GBM is another vital consideration when attempting to model TME conditions *in vitro*. These consist of immune cells, including invading macrophages and resident microglia, stromal cells including astrocytes, vascular endothelial cells and pericytes (88). Microglia and astrocytes, both of which are found in abundance in the brain, accounting for approximately 30-50% and 50% of the volumes in the tumour and brain tissue, respectively (89, 179) have been implicated in glioma invasion and survival (88) highlighting the importance of their inclusion in comprehensive studies of the effects of TME on GBM. For a more indepth review refer to Decordova et al., 2020 (180). Throughput is a significant limitation on the extent to which the effects of microglia and astrocytes can be utilized; however, several academic laboratories have generated 3D co-culture models to elucidate the roles of these cell types that offer potential for adaption to a higher throughput format. One such study has generated luciferase reporter GBM cell lines with a cell viability readout on 3D floating co-culture spheroids of bioluminescent GBM tumour cells and non-luminescent rat astrocytic cells. The research demonstrated an astrocyte-mediated increase in TMZ resistance in four of a panel of six GBM tumour cell lines (181). This *in vitro* model was also successfully employed along with stem cells and *in vivo* models to demonstrate growth-supportive effects of astrocytes (182). The protective effects of astrocytes in co-culture against the main chemotherapeutics, TMZ and vincristine, have also been observed in subsequent research in 2D and 3D co-culture and demonstrated to be contact-dependent (183, 184) where drug resistance might be due to the transfer of mitochondria through tunnelling nanotubes connecting with and rescuing the tumour cells (183). Colleagues from this laboratory utilized the same 3D HA-based model with co-cultured microglia to demonstrate chemoresistance that was not present in monocultures of GBM cell lines (185).

When designing an optimized platform for high throughput combinatorial screening, balancing the need for larger screening libraries to generate novel drug candidates and physiologically relevant models to better predict drug response is crucial. In order to better model tissue-like features and cellular interactions of the TME, recent HTS efforts have utilized 3D *in vitro* models of GBM. High content screening against a panel of 83 chemotherapeutics in both 2D and 3D cultures in the U87 cell line highlighted shortcomings in the former’s ability to detect drug sensitivities with only those resulting from 3D cultures aligning with *in vivo* results (138). These findings are also supported by an independent study demonstrating TMZ sensitivity is decreased in 3D cultures of the common GBM cell lines, U87, U251, and SNB19, when compared with its 2D counterpart (178) Others have employed a 3D *in vitro* model with cell lines cultured in a HA-gelatin based hydrogel to model the ECM in 3D cultures, with chondroitin sulphate, chitosan and collagen/gelatine have also been utilized (186). Increased drug resistance was attributed to the co-cultured microglia and astrocytes.

Finding the appropriate balance in a GBM model between the complexity of the TME and the practical considerations of HTS is a challenge that has to be addressed for more successful translation of hits in drug discovery. In recent research, this balance may well have been achieved (187) in a study of 461 mostly FDA-approved drugs in 12 patient-derived GSCs, that incorporated both a 2D culture model in the primary HTS followed by a secondary 3D model for hit validations experiments. Results from this study demonstrate significant differences in sensitivity to hits representing multiple drug classes across patient-derived GSC models.

## The implications of rational combinatorial small compound drug screening in GSC

In complex heterogeneous tumours, no single molecular event drives continued proliferation and tumour progression, and redundancy in signalling pathways limits the efficacy of therapies targeting single pathways (188). Network and pathway switching permit rapid tumour evolution and therapeutic evasion; this requires a holistic approach to understand cancer cell signalling networks, ‘driver’ pathways, and how best to collapse the robustness of such networks to address therapeutic resistance. Thus the complexity of GBM has been likened to the “three lock problem”, describing that a door with three locks will not open any better even if keys are found to one or two locks (106). With so many different signalling pathways and cellular heterogeneity involved in tumour growth, combinations of targeted agents may well be most effective in treating rapidly evolving heterogeneous tumours, provided we can identify the network changes that permit cancer cells to subvert single agents.

In addition to the importance of the disease model and optimal drug combination delivery, equally relevant questions are; what is the rationale behind choosing specific drugs for potential combination therapies? How many drugs will be most beneficial to patients? What is the quickest and most successful way to identify these combinations? The rationale behind many drug combinations strategies tested in clinical trials studies is often unclear, and to date, unsuccessful. Even with the automated HTS instruments, exhaustive screening of drug combinations becomes quickly impractical, both in terms of time and cells required, as the number of potential drug and dose combinations increases exponentially with the number of tested drug components and dose levels. This combinatorial explosion requires intelligent computational-experimental strategies to guide the discovery of the most potent and less toxic combinations to be prioritized for further preclinical development before entering into lengthy and costly animal or clinical studies (189).

For rational combinatorial screening, there is a need for computationally efficient and experimentally feasible approaches that can (i) reduce combinatorial search space of potential combinations, (ii) and to identify both synergistic and safe combinatorial therapies for each individual patient and cell-context. For cancer-selectivity, it is important to guarantee that the combinations target stem cells and bulk tumour cells in GBM, while avoiding co-inhibition of non-malignant cells, to avoid treatment resistance and severe toxic effects. Ianevski et al. demonstrated recently the feasibility of using XGBoost machine learning (ML) algorithm together with ex vivo single-agent responses and single-cell transcriptomic profiling to identify patient-specific and cancer-selective pairwise combinations for treatment-refractory AML patients, each with different molecular backgrounds and synergy mechanisms (190). Using flow cytometry drug assay, they demonstrated that the predicted combinations resulted not only in synergistic cancer cell co-inhibition, but were also capable of targeting specific AML cell subpopulations that emerge in differing stages of disease pathogenesis or treatment regimens. Such patient- and cell subpopulation-specific combinatorial approaches may avoid overlapping toxic effects through co-inhibiting mainly malignant cell types, therefore increasing their likelihood for clinical success.

There are many experimental and computational challenges that need to be solved to implement similar translational approaches for GBM combinatorial screening. Machine learning algorithms could help in predicting full dose-response matrices using a minimal number of combinatorial experiments, thereby enabling more cost-effective, yet systematic combinatorial screens (191). Computationally, one needs to consider also higher-order combinations of more than two drugs to facilitate the current paradigm shift from the traditional ‘two drugs in combination’ to more complex ‘multi-drug cocktails’ (192). While there are a number of machine learning models for prediction of pairwise drug-dose combinations (193), accurate prediction of higher-order combination effects with more than two drugs remains an unsolved problem. A tensor learning model that enables the accurate prediction of pairwise dose-response combinations for each cancer cell line (194) has been recently developed. Higher-order tensor learning may enable generalising and learning from the currently still rather limited combination data to explore and score in an iterative manner so-far untested massive drug spaces of higher-order combinations (195), hence enabling adaptive learning of best combinations for each cell context or patient individually.

## Outlook and conclusions

At the time of writing, a search for the terms ‘glioblastoma’ and ‘combination’ on the clinicaltrials.gov website returns 604 separate clinical trials; 29 not yet recruiting, 123 recruiting, 65 active but not recruiting, 102 suspended, terminated or withdrawn, 40 with status unknown and 242 completed. Despite the many successes of modern drug discovery strategies across several cancers, such approaches have yet to provide significant benefit for the majority of GBM patients (196). As detailed in this review, this failure can be attributed to several factors, including remarkable heterogeneity of GBM between and within individual patients, multiple redundant and adaptive pathway signalling mechanisms allowing GBM to escape from any substantial consequence of individual target perturbation, poor preclinical models, which fail to recapitulate the complex microenvironment and pathophysiology of clinical GBM. Below we outline some of the key technological advancements which are well placed to address past failures and maximize future opportunities of targeted therapies and their combinations in GBM.

### Improving GBM high throughput screening

To date, hypothesis-driven or targeted drug discovery such as efforts to inhibit the receptor tyrosine kinase (RTK) signalling pathways have been largely disappointing. Overexpression or mutations of EGFR occur in ~50% of GBM, yet clinical trials targeting this RTK in combination with standard care or other therapies have proven ineffective for GBM treatment (197). If this is to be overcome, future drug discovery programs must include combined targeted phenotypic approaches with comprehensive screening strategies incorporating phenotypic readouts as the primary HTS and target-based secondary and tertiary assays.

GBM cell lines have been integral to many HTS efforts in the past but are subject to significant genetic drift. Analysis of one widely used line, U87MG, has demonstrated that following 50 years of culture, it no longer reflects the phenotype of its tumour of origin (198). By employing GSC isolated from primary human tissue samples and maintained at low passage numbers, phenotypic screening can provide a more accurate representation of the tumour cell physiology than observed in cell lines (199). In addition, high throughput screens can incorporate the genetic diversity of GBM tumours by utilising GSCs from the main tumour-intrinsic transcriptional subtypes: Classical, Mesenchymal and Proneural as defined by aberrations and gene expression of EGFR, NF1, and PDGFRA/IDH1 (70).

GSC are highly resistant to chemotherapy and radiation (200). The acquisition of resistance to the SOC treatment in GBM is one of, if not the most significant hindrance to any new therapeutic and must be addressed in any cell models of GBM to be used for drug discovery. In primary high throughput screens, the rapid reformation of highly resistant tumours following surgical resection can be modelled by generating both chemotherapeutic and radioresistant GSC lines. As we have suggested, the most likely advances in GBM treatment will originate from the combination of additional therapeutic drugs with the existing SOC, so investigating new combinations of treatments/drugs in screening paradigms including irradiation and TMZ is absolutely critical to success.

Additionally, the hypoxic microenvironment plays a central role in the malignancy of GBM, acting on gene expression (201), promoting angiogenesis (202) and is implicated in tumour metastasis (203). The hypoxic environment presents further obstacles to the success of HTS by the generation of chemoresistance through various means, including activation of HIF1α and inducing radioresistance by preventing the formation of reactive oxygen species, the primary mechanisms of ionising radiation (204). The majority of drug discovery efforts in GBM have not factored in the significant contributions this environment plays on therapy resistance. Research in a 3D *in vitro* model has demonstrated the potentiation of TMZ resistance due to hypoxic conditions in GBM cell lines (178). However, unlike their 3D counterparts, 2D *in vitro* cell models are unable to foster an oxygen-deprived TME. This limitation can be overcome in future HTS programs by employing GSC cultures that are maintained, concurrently, in normoxia and hypoxia, through the use of standard incubators and physiological cell culture workstations, respectively.

Finally, the significant financial implications of running large HTS programs as is the norm in the pharmaceutical industry make these strategies not feasible for non-profit organisations in academic research. Rather, to achieve the same outcomes, anti-cancer targeted small molecule libraries and repurposed drugs can be utilized. This focused approach has the combined advantages of providing a more direct path to clinical translation by using existing compounds with known safety profiles and by providing molecular detail on the aetiology of the disease.

### Application of more predictive preclinical models in the GBM screening cascade

*In vivo* models of GBM are used in the advanced stages of drug discovery; they are expensive and not feasible for high-throughput testing of candidate drugs and drug combinations. Genetically engineered mouse models have a low incidence rate and long latency, while implanted tumours grow expansively due to high proliferative rates, with minimal diffuse local invasion when compared to clinical GBM. For this reason, there is a need for more relevant preclinical models which recapitulate the TME and can be incorporated downstream of high throughput screens to validate hit compounds. Due to the complexity of GBM, cell response and behaviour are vastly different when comparing a 3D microenvironment with traditional 2D monolayer cell culture models. This is particularly important when considering mechanisms of tumour cell invasion, which is a major cause of treatment failure in GBM, contributing to poor prognosis. Therefore, we suggest that a combination of complementary 3D assays be used to collectively assess the cell-ECM interaction and invasion types (into astrocyte rich stroma and along blood vessels/white matter tracts), which are observed *in vivo*.

3D tumour spheroid-based functional assays which aim to mimic the *in vivo* like invasion patterns, preferentially migrating along basement membranes (blood vessels and white matter tracks). While it is not possible to replicate all aspects, 3D assays may be used to recapitulate the defining aspects of GBM invasion/migration (205, 206). To mimic 3D invasion, spheroids submerged in a reconstituted basement membrane matrix, such as growth factor reduced Matrigel, can be used to determine the invasive potential of a cell line and support screening for compounds and/or drug combinations that may inhibit invasion (207). While Matrigel is a viable matrix, it is important to note the high percentage of collagen and reproducibility issues with batch-to-batch variability. Radial migration of spheroids placed onto a basement membrane such as laminin or Matrigel with media substituted with hyaluronan may be used to recapitulate perivascular glioma cell migration. Typically, GBM invasion/migration occurs along a basement membrane rather than through one, such as with the Boyden chamber membrane assay.

Recapitulating all aspects of the brain microenvironment is difficult *in vitro*. Therefore, ex vivo brain slice assays are used to maintain the complexities of the extracellular matrix and brain architecture and preserve *in vivo* morphology on which to grow tumour cells or spheroids and monitor cell behaviour in response to treatment (208–210). It has been shown that GBM stem cell motility decreases compared to 2D culture when maintained in this way, presumably due to additional barriers such as the extracellular matrix and the need for extracellular matrix remodelling (211). GBM cells also proliferate and differentiate differently depending on which region of the coronal brain slice the cells are injected (212). Surgically resected human tumour slices may also be used for ex vivo drug screening to personalize cancer therapy (213). The analysis of tissue slices is often by histochemistry, but recently, whole transcriptome sequencing of resected human GBM tumour slices has been performed to determine gene expression differences after drug treatment (214). Further advances in automated high-content confocal imaging enable systematic testing of candidate drugs and drug combinations on phenotypic markers in advanced 3D *in vitro* and ex-vivo model systems. Organotypic brain/tumour slice assays may play a useful role in bridging the gap between existing *in vitro* and *in vivo* assays, especially when investigating tumour cell migration, invasion and growth to evaluate novel therapeutic strategies.

### Cost-effective and transparent AI-guided prediction of drug combinations

Large-scale multi-dose combinatorial screening requires extensive resources and instrumentation beyond the capability of most academic laboratories. Testing of thousands of drug-dose combinations is also impossible in limited numbers of primary cells from patients. The development of transparent and robust experimental and computational strategies is expected to lead to effective prioritisation and validation of optimal drug combination and dose ratios toward next-generation preclinical and clinical testing platforms. The predictive models, based on both physiologically relevant GBM conditions and practically feasible ML models, are therefore expected to lead to both cost- and time-efficacy in academic cancer research. Using such strategies, the drug screening efforts can be targeted to verifying the most promising drug combinations, with maximal cancer-selectivity, thereby significantly accelerating the future design and testing of combination therapies, as well as increasing the likelihood of their success in preclinical and clinical studies with the aim to improve both combination efficacy and tolerability (215).

Many of the most accurate ML or artificial intelligence (AI) models are not transparent for humans, e.g., those based on deep learning or tensor learning algorithms, and they may rely on an overly large set of input features for cost-efficient implementation. For widespread adoption among experimental researchers, the learning algorithms need to be transparent and explainable to experimental researchers, including a clear description of the optimisation objectives (synergy, efficacy and/or toxicity) and quantitative performance and confidence evaluation (e.g. using conformal prediction), which help the experimentalists to decide when and how to use the algorithms to obtain valid results (216). For experimental feasibility, there is also a need to implement effective computational approaches that make use of partial measurements of the full drug-dose spaces to predict the most potent higher-order combinations and to provide high-resolution information of response landscapes across various dose combinations, critical for clinical translation (e.g. low dose and less toxic synergies).

The increased understanding of disease heterogeneity and new emerging methodologies described in this review article enable modern non-reductionist and more evidence-led discovery strategies which embrace the complexity of GBM. We believe such approaches will facilitate a more systematic and transparent approach to the identification and prioritisation of new drug combinations which can contribute to improved treatments for GBM patients.

## Author contributions

DE conceived the review, wrote the manuscript and is the corresponding author. TJ is first author and wrote the manuscript. LM compiled the clinical trials tables and wrote the manuscript. JS compiled the clinical trials data. SE, GH and MF contributed to writing the manuscript and provided critical editing. TA and NC wrote the manuscript. All authors contributed to the article and approved the submitted version.
